# Evaporative coolers and wildfire smoke exposure: a climate justice issue in hot, dry regions

**DOI:** 10.3389/fpubh.2025.1541053

**Published:** 2025-02-26

**Authors:** Gina M. Solomon, Nayamin Martinez, Julie Von Behren, Isabella Kaser, David Chang, Aditya Singh, Stephanie Jarmul, Shelly L. Miller, Peggy Reynolds, Mohammad Heidarinejad, Brent Stephens, Brett C. Singer, Jeff Wagner, John R. Balmes

**Affiliations:** ^1^School of Medicine, University of California, San Francisco, San Francisco, CA, United States; ^2^Central California Environmental Justice Network, Fresno, CA, United States; ^3^Department of Epidemiology and Biostatistics, University of California, San Francisco, San Francisco, CA, United States; ^4^Tracking California, Public Health Institute, Oakland, CA, United States; ^5^Department of Civil, Architectural, and Environmental Engineering, Illinois Institute of Technology, Chicago, IL, United States; ^6^Office of Environmental Health Hazard Assessment, Oakland, CA, United States; ^7^University of Colorado Boulder, Boulder, CO, United States; ^8^Lawrence Berkeley National Laboratory, Berkeley, CA, United States; ^9^Environmental Health Laboratory Branch, California Department of Public Health, Richmond, CA, United States; ^10^Division of Environmental Health Sciences, School of Public Health, University of California, Berkeley, Berkeley, CA, United States

**Keywords:** indoor air quality, PM2.5, farmworker, Hispanic, climate change, California, swamp cooler

## Abstract

Low-income families in dry regions, including in the Southwestern United States, frequently cool their homes with evaporative (“swamp”) coolers (ECs). While inexpensive and energy efficient compared to central air conditioners, ECs pull unfiltered outdoor air into the home, creating a health hazard to occupants when wildfire smoke and heat events coincide. A community-engaged research project to reduce wildfire smoke in homes was conducted in California’s San Joaquin Valley in homes of Spanish-speaking agricultural workers. A total of 88 study participants with ECs were asked about their level of satisfaction with their EC and their willingness to pay for air filtration. About 47% of participants reported dissatisfaction with their EC, with the most frequently reported reason being that it brings in dust and air pollution. Participants were highly satisfied with air cleaners and air filters that were offered to them free-of-charge. However, a willingness to pay analysis showed that air filtration solutions would not be adopted without significant subsidies; furthermore, air filtration would be an ongoing cost to participants due to the need to regularly replace filters. Short-term filtration solutions for EC users are feasible to implement and may reduce smoke exposure during wildfire events. Such solutions would need to be offered at low-or no-cost to reduce barriers to adoption. Longer term solutions include prioritizing homes with ECs in wildfire smoke exposed regions for replacement with air cooling technologies that provide clean air. Because ECs are disproportionately in low-income homes, addressing smoke intrusion through these devices is an environmental justice issue.

## Introduction

Severe wildfires across the globe have increased due to climate change, resulting in heat, drought and high wind events, which exacerbate fires (1, 2). Wildfire smoke now accounts for half of ambient particulate matter under 2.5 microns in diameter (PM_2.5_) in the Western U.S. compared to <20% a decade ago (3). Exposure to wildfire smoke is associated with global increases in all-cause mortality, respiratory and cardiovascular mortality, with an estimated total of 52,480–55,710 premature deaths attributable to wildland fire PM_2.5_ in California over the 11-year period from 2008 to 2018, and an estimated economic impact during that time of $432 to $456 billion (4, 5). Climate change is expected to increase wildfire risks, with the number of premature deaths attributable to fire-related PM_2.5_ projected to double by the end of the century in the contiguous United States (6, 7).

In hot and dry regions of the world, some homes have evaporative (“swamp”) coolers which may be in use during smoke events because wildfires tend to occur during hot, dry periods. Evaporative coolers (ECs) are common in low-income households because they are reasonably effective at cooling while costing about half as much and using about one-quarter the energy as central air-conditioners (8). The global EC market has been estimated at $6.7 billion in 2022 and is projected to reach $10.8 billion by 2032 with most of the EC technology deployed in residences. Growth projections are due to warming temperatures and increased need for affordable cooling technologies in multiple regions including the Western United States, China, and India (9).

ECs are used in the San Joaquin Valley (SJV) of California, especially in the homes of farmworkers and other low-income residents. The SJV is surrounded by mountain ranges, and due to local meteorologic conditions, an inversion layer frequently forms, trapping wildfire smoke and other air pollution in the region, resulting in the worst year-round particulate matter pollution in the U.S. (10). A 2019 California Energy Commission survey found that 12% of single-family homes overall in California, and 27% of mobile homes, are cooled by ECs, with use clustered in the SJV (11). ECs use a fan to draw large amounts of outdoor air through moist pads, typically made of cellulose or aspen wood, into the home. The air is cooled as heat is removed and water from the pads evaporates. The cooler air mixes with the hotter, drier air in the home to reduce the overall temperature (and raise the humidity). The resulting mixture is pushed out through windows and cracks.

Although ECs have been in use for decades, there has been little research on their effects on indoor air quality and their performance during wildfire smoke events. EC pads have relatively low filtration efficiency for particulate matter, and there are no high efficiency filtration options commercially available. Specifically, Paschold et al. (12) reported that the passage of air across moist EC pads reduced particulate matter under 10 microns in diameter (PM_10_) by up to 50%, and PM_2.5_ by 10–40% in a laboratory setting (12). A field study in 10 Texas homes over 4 days under normal (non-wildfire) conditions reported a reduction by approximately 40% of outdoor PM_10_ and 35% of PM_2.5_ (13). A 35% reduction in PM_2.5_ during periods of wildfire smoke that may exceed 250 μg/m^3^ means that indoor PM_2.5_ concentrations would still exceed 150 μg/m^3^ – unsafe levels well above regulatory limits. In addition to PM_2.5_ infiltrating homes, ozone, metals, polycyclic aromatic hydrocarbons (PAHs), and volatile organic compounds (VOCs) may also be found (14, 15). Recently there has been interest in understanding the filtration and operation of ECs in the US (16, 17).

In the summer of 2020, wildfire smoke from the Santa Clara Unit Lightning Complex Fire, the Caldor Fire, the Creek Fire, and the August Complex Fire all converged on the SJV, creating hazardous smoke conditions combined with high heat for many weeks (18). The Central California Environmental Justice Network (CCEJN), a community-based organization serving agricultural workers, received reports from many SJV residents with health concerns due to the excess smoke and heat. Community members reported that they could not breathe well in their homes due to the smoke if the EC was operating, but that the heat was intolerable without the use of the cooler. Many agricultural workers have children with asthma and elders with chronic cardiopulmonary disease under one roof and could not keep their families safe from both the heat and the smoke. Community members asked if any filtration was available to protect them from smoke exposure in their homes during wildfires. Development of an effective and affordable filtration technology for ECs would enable continued use of existing equipment while delivering well-filtered air into homes of this vulnerable population.

## Methods

### Study site

**Figure 1:**
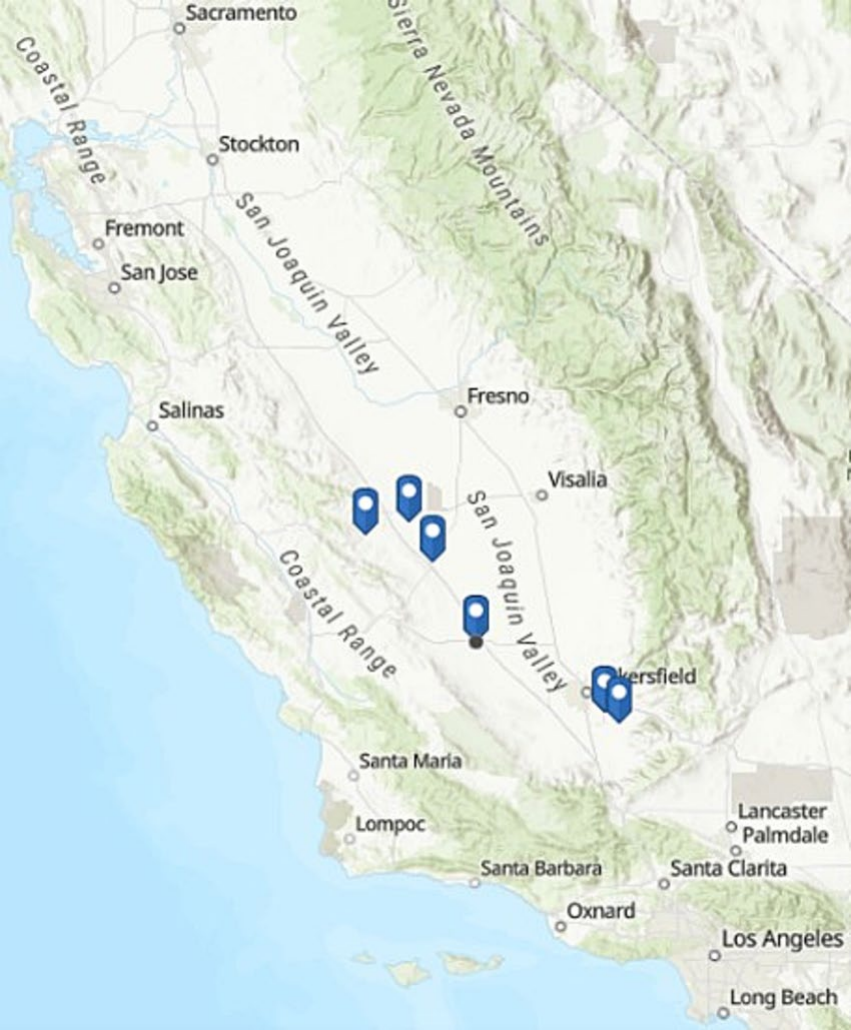
Map of the FRESSCA study locations, California, 2022–2023. Accessed using Google Maps.

The Filtration for Respiratory Exposure to wildfire Smoke from Swamp Cooler Air (FRESSCA) study was an air quality experimental intervention study conducted from 2021 to 2023 in Fresno, Kings, and Kern counties in California’s SJV, a low-income agricultural area (Figure 1). This region has long, hot, dry summers with average daily maximum temperatures over 32 degrees Celsius (90 degrees Fahrenheit) (19). In 2021, the National Weather Service recorded more than 67 days above 37 degrees Celsius (100 degrees Fahrenheit) in Bakersfield, CA (Kern County) (20).

### Measures

The FRESSCA project included community engagement, development of filtration strategies for ECs, and air quality monitoring inside homes to test various interventions. The study also included questionnaires about health status, home comfort, satisfaction with the EC and the filtration strategy, and a question about willingness-to-pay for effective filtration. The development and laboratory testing of the interventions is described elsewhere (21), and the indoor air monitoring data analysis is ongoing. Here we focus on findings related to participants’ health status, home comfort, satisfaction with the intervention, and willingness-to-pay for indoor air with reduced wildfire smoke.

### Development of filtration interventions

As described elsewhere (21), prototype filtration solutions were developed and initially tested in the laboratory using the concept of do-it-yourself (DIY) box fan filters, based on the Corsi-Rosenthal published design, as an inspiration (22). DIY strategies can provide an affordable and effective solution to reducing exposure to wildfire smoke (23).

The solution that best balanced effective filtration with minimum reductions of airflow to ensure sufficient cooling was 4-inch MERV 13 filters. The filters were strapped to the exterior air intakes of ECs using bungee straps, with gaps taped to improve the seal as necessary. Ideally the MERV 13 filters also may include impregnated carbon or other sorbent media to filter VOCs. This solution was envisioned to last throughout a wildfire smoke event lasting days or weeks.

To compare interventions and also address indoor sources of PM_2.5_, we tested multiple interventions alone and in combination in our field study, including: The MERV 13 filter solution, an indoor portable high-efficiency particulate absorbing (HEPA) air cleaner, and a box fan filter (pilot phase only). The box fan filter was dropped from the study in Year 2 due to expressed participant preference for the HEPA air cleaner, the observation that participants rarely used the box fan filter, and space limitations in many homes (the box fan filter was significantly larger than the HEPA air cleaner and could not be located next to a wall).

### Participant recruitment

To test the intervention in homes, we recruited a total of 88 participants (31 in the pilot phase in 2022 and 57 in the intervention phase in 2023). Participants were recruited by our community partner, CCEJN, through posting of flyers, social media, a recruitment video, word-of-mouth, and door-to-door efforts in the local community. In both phases, participation was restricted to individuals living in non-smoking homes to minimize other sources of PM_2.5_ in the study homes. In the pilot year, both men and women were participants and responded to the questionnaires; in the second year, we included additional study aims and measurements relevant to wildfire smoke and breast cancer risk, so the questionnaire respondents in 2003 were all women.

### Testing of filtration interventions

**Figure 2:**
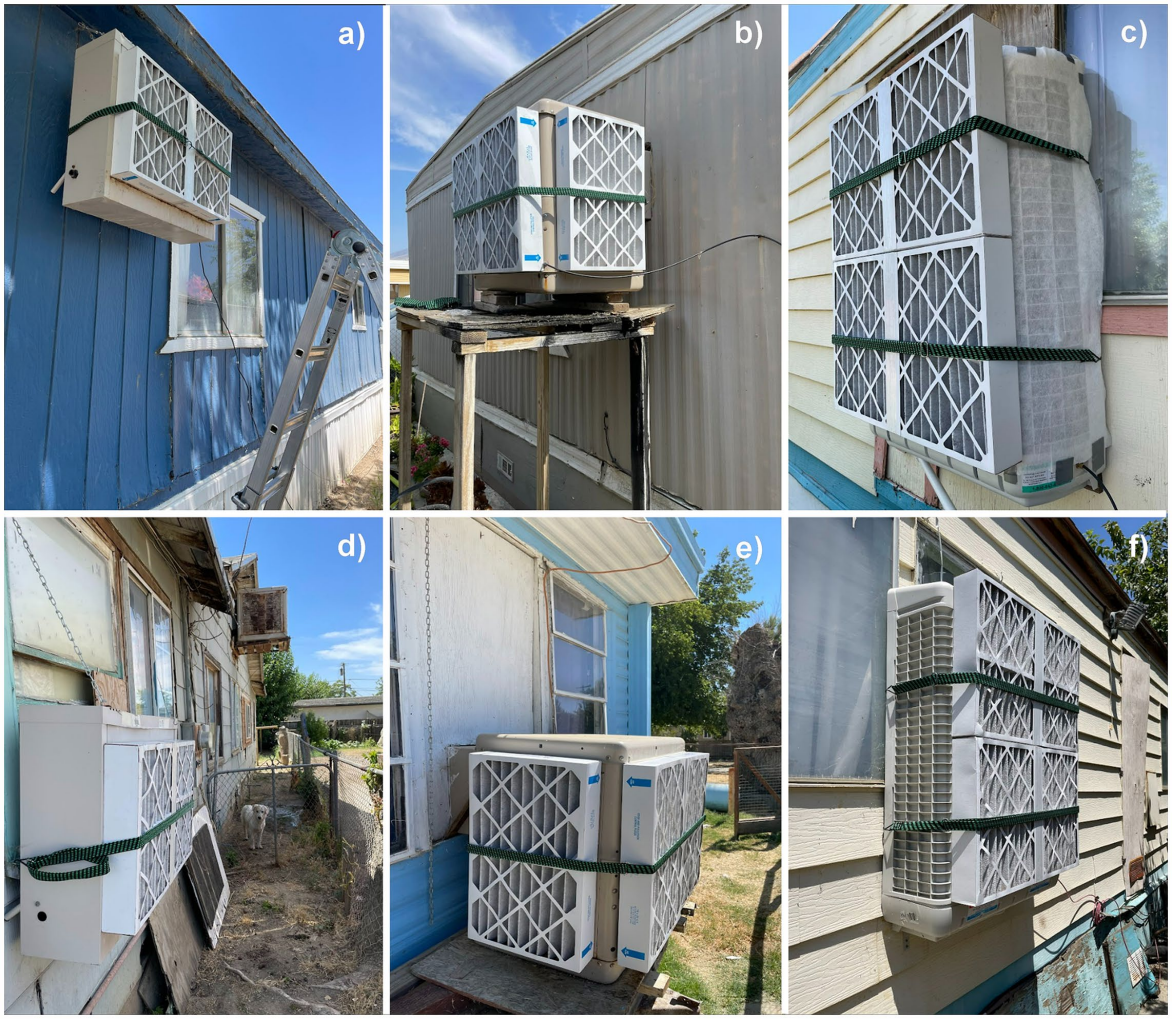
Evaporative Coolers in the study region with our DIY Filter Interventions: **(a, d)** are single intake ECs, **(b, e)** are 3 side intake ECs, **(c, f)** are curved side intake ECs in which **(f)** already had the sides blocked and for **(c)** we used a hybrid solution of Sheet Filter media with our DIY Filter solution.

Community members who consented to participate received free professional servicing to ensure their EC was working properly and the participant in each home received gift cards to compensate for their time spent and the inconvenience of having equipment and study staff in their homes. The study was approved by the Public Health Institute IRB # I22-002 and #I22-002a. A total of 55 participant homes received HEPA indoor air cleaners and 36 participant homes also had filters installed on the EC by our study team (Figure 2).

### Participant questionnaires

Participants responded to questionnaires in both the spring before deployment and the fall after deployment of the filters to assess thermal comfort, experiences with smoke, satisfaction with the EC and with the study interventions, and other factors that might indicate willingness to use filtration in the future. Participants also completed a modified version of the St. George’s Respiratory Questionnaire (SGRQ), a validated instrument for measuring health-related quality of life based on assessment of respiratory symptoms, activity limitations, and psychosocial effects in the prior 3 months (24). Questions were also asked about age, sex, type of work, length of time living in home, home ownership, and current insurance status. Questionnaires were available in English and Spanish and were administered by study staff, who recorded the responses in Qualtrics (Qualtrics, Provo, UT).

### Data analyses

The data with questionnaire responses was exported from Qualtrics and analyzed using SAS version 9.4 (SAS Institute Inc., Cary, North Carolina). For age of the participants, we calculated the mean in years. All other variables were categorical and we examined the frequency distributions by responses.

## Results

### Participant characteristics

Participants from both study phases all identified as Hispanic/Latinx and primarily spoke Spanish at home (Table 1). The average age of the 2023 participants was 43 years (range 22–61). Eighty-three participants identified as female and five as male. Most of the participants were agricultural workers (64%) or working in food packaging or processing (17%), shown in Table 1. About two-thirds (65%) of participants had lived in their home for more than 5 years and 69% owned their home. Most of the participants (72%) received health insurance through Medi-Cal, 7% had private insurance and 21% were uninsured.

**Table 1:** FRESSCA study participant characteristics and survey responses before air filter intervention, California, 2022–23.

	*N*^a^ (%)
Total participants	88 (100)
Average age in years	43 (range 22–61)
Sex	
Female	83 (94.3)
Male	5 (5.7)
Type of work	
Agricultural	56 (63.6)
Food packaging or processing	15 (17.0)
Family member of farmworker	6 (6.8)
Other types of work or retired	7 (8.0)
Not working or not specified	4 (4.5)
Length of time living in home^b^	
<1 year	2 (3.5)
1–5 years	18 (31.6)
More than 5 years	37 (64.9)
Home ownership or rental	
Own home	61 (69.3)
Rent home	26 (29.5)
Unknown	1 (1.1)
Type of health insurance^b^	
Medi-Cal	41 (71.9)
Private insurance	4 (7.0)
Uninsured	12 (21.1)
Outdoor air quality rating	
Extremely bad	36 (40.9)
Somewhat bad	25 (28.4)
Neither good nor bad	20 (22.7)
Somewhat good or excellent	4 (4.6)
Satisfaction with summer indoor air quality	
Very dissatisfied	32 (36.4)
Somewhat dissatisfied	16 (18.2)
Neutral	25 (28.4)
Somewhat satisfied	6 (6.8)
Very satisfied	9 (10.2)
Common reasons not satisfied with summer indoor air quality^c^	
Dust	62 (70.5)
Odors	60 (68.2)
Air pollution from outdoors entering home	54 (61.4)
Frequency home is too hot in summer	
Every day	50 (56.8)
Few times a week	19 (21.6)
Few times a month	4 (4.5)
Few times a year	6 (6.8)
Never	9 (10.2)
Satisfaction with evaporative cooler	
Very dissatisfied	24 (27.3)
Somewhat dissatisfied	17 (19.3)
Neutral	21 (23.9)
Somewhat satisfied	11 (12.5)
Very satisfied	13 (14.8)
Common reasons not satisfied with evaporative cooler^c^	
Brings in dust, odor, and/or air pollution from outdoors	50 (56.8)
Not effective or does not cool enough	32 (36.4)
Too noisy	28 (31.8)
Uses too much energy	25 (28.4)
Most willing to pay for a filter that reduces exposure to smoke	
<$20	65 (73.9)
$21–$50	7 (8.0)
$51–$100	5 (5.7)
More than $100	7 (8.0)

^aTotals vary due to missing or unknown responses, some questions only asked in second year of survey. bQuestion not asked in pilot phase for N = 31 participants. cMore than one reason listed for some participants.^

### Air quality perceptions

Prior to the EC filter and HEPA indoor air cleaner interventions, most participants (69%) reported that the outdoor air quality where they lived and worked was “extremely bad” or “somewhat bad” (Table 1). The majority of participants (55%) were not satisfied with their indoor air quality in the summer months. About 57% of participants reported that the home was too hot during the summer. Study participants had mixed responses to questions about satisfaction with their EC. About 47% of participants reported being dissatisfied with their EC. The most common reasons reported for dissatisfaction for both indoor air quality at home and ECs were dust, odors, and outdoor air pollution.

Study participants were highly satisfied with the filtration solutions deployed by the study intervention, including the HEPA indoor air cleaner and the EC filters. When some of the participants were surveyed again in the fall after the summer months with the air filters installed, about 80% of the participants said that they were very or somewhat satisfied with the EC air filters provided by the study (21 out of 26 participants who received the EC filters, Table 2). The reported level of satisfaction was even higher for the HEPA indoor air cleaner that was provided, with 96% saying that they were very or somewhat satisfied with it (*N* = 46 participants, Table 2). Although participants were generally not satisfied with the air quality inside the home and were satisfied with the air filtration, most of them (74%) reported that they were willing to pay <$20 for an air filter that would reduce exposure to wildfire smoke (Table 1).

**Table 2:** FRESSCA participants’ reported satisfaction with filters after study completion (intervention phase), California, 2023.

	*N* (%)
Total participants	46 (100)
Level of satisfaction with EC air filters (not provided to all homes)	
Somewhat Dissatisfied	1 (2.2)
Neutral	4 (8.7)
Somewhat satisfied	2 (4.3)
Very Satisfied	19 (41.3)
Did not have EC filter or did not answer	20 (43.5)
Level of satisfaction with HEPA indoor air cleaner (provided to all homes)	
Somewhat dissatisfied	1 (2.2)
Neutral	1 (2.2)
Somewhat satisfied	9 (19.6)
Very satisfied	35 (76.1)

### Health and respiratory symptoms

Almost half (47%) of study participants reported their health as “fair” or “poor” (Table 3). This percentage is higher compared to 24.5% of Hispanic adults reporting “fair” or “poor” health in the 2022 California Behavioral Risk Factor Surveillance System report (25). Although most participants (63%) reported that they had not coughed in the past 3 months or had only coughed with respiratory infections, about 18% reported coughing several days a week or every day. Most participants said that their respiratory problems did not affect their ability to work (79%), although about 13% reported that respiratory symptoms interfered with their job, made them change jobs, or caused them to stop working.

**Table 3:** FRESSCA study participant survey responses about respiratory health, California, 2022–2023.

	*N*^a^ (%)
Total participants	88 (100)
Describe current health	
Very good	6 (6.8)
Good	40 (45.5)
Fair	38 (43.2)
Poor	4 (4.5)
Respiratory problems over past 3 months: coughed	
Not at all	37 (42.0)
Only with respiratory infections	18 (20.5)
A few days a month	16 (18.2)
Several days a week	8 (9.1)
Almost every day	8 (9.1)
Respiratory problems over past 3 months: shortness of breath	
Not at all	48 (54.5)
Only with respiratory infections	13 (14.8)
A few days a month	16 (18.2)
Several days a week	4 (4.5)
Almost every day	6 (6.8)
Respiratory problems over past 3 months: respiratory attacks	
None of the time	72 (81.8)
One time	8 (9.1)
Two or more times	7 (8.0)
Describe respiratory condition	
The most important problem I have	4 (4.5)
Causes me quite a lot of problems	2 (2.3)
Causes me a few problems	19 (21.6)
Causes no problems	58 (65.9)
Respiratory health impacts on ability to work	
Respiratory problems made me stop working completely	9 (10.2)
Respiratory problems interfere with my job or made me change my job	2 (2.3)
Respiratory problems do not affect my job	70 (79.2)
Did not respond or did not work	7 (8.0)

^aTotals vary due to missing or unknown responses.^

## Discussion

Climate vulnerability studies have often neglected farmworkers and the environmental and public health impact on these communities (26). Farmworker communities are already disproportionately experiencing the impacts of climate change due to flooding, drought, excessive heat, and wildfire smoke impacts (27). Ambient exposures to wildfire smoke and poor air quality may be difficult to mitigate for outdoor workers (28). Indoor exposures, however, should be feasible to mitigate, offering people respite during non-working hours. Central air conditioning systems with filtration are rare in farmworker and low-income homes in the Southwestern United States (16). This project attempted to address farmworker concerns by developing, deploying, and testing an intervention to address wildfire smoke intrusion through ECs, thereby addressing an environmental justice concern.

Despite the relatively young average age of our study population, the fact that most people were actively employed, and that all participants were non-smokers, it was concerning that self-reported health was only fair or poor in nearly half of our participants. Environmental exposure concerns and lack of access to health care are potential contributing factors. Rates of respiratory symptoms were relatively low in the population, with most participants responding “no” to cough, shortness of breath, or respiratory attacks over the past 3 months, except in the setting of respiratory infections. Despite exposure to poor air quality, this population might report low rates of respiratory symptoms because they are relatively young, non-smoking, and physically active workers, which may lead them to believe they are healthy. However, it is notable that about 13% of our study participants reported that respiratory symptoms interfered with their job, made them change jobs, or caused them to stop working.

The intervention study was challenged by multiple factors, including the wide diversity of EC shapes and sizes requiring customization of the filter solution; hazards associated with attaching filtration to rooftop ECs; and the fact that the fans in ECs are designed to compensate for only a small amount of airflow resistance. We found that affordable and readily available filters such as MERV-13 1-inch filters severely disrupted the airflow through the EC, thereby interfering with cooling (21). Our DIY-filtration solution required at least a 4-inch MERV 13 filter in order to achieve desired level of filtration without drastically restricting airflow. Unfortunately, the 4-inch filters are not readily available in local stores, and they are considerably more expensive. Indoor filtration at the outflow of the EC was not feasible both due to the fan weakness described above and the high moisture content of the outflow air, so multiple filters were needed to cover the exterior surface of the EC (Figure 2).

During our pilot and intervention phases, which lasted approximately from July–October for 2 years (2022–2023), there was no significant local impact from a wildfire smoke event, making it challenging to discern the quantitative air quality impacts of our solutions (21). Despite these challenges, participants noticed a subjective improvement in indoor air quality with our filtration interventions, potentially associated with decreased intrusion of dust and other large particles. Many participants requested to keep the EC filters or get additional filters at the end of the study; participants kept the HEPA indoor air cleaners and were provided with new HEPA filters after the study ended.

Unfortunately, we did not identify a filtration solution that aligns with the willingness-to-pay expressed by the study participants. Only about a quarter of study participants were willing to pay more than $20 for an air filtration solution and only 7% were willing to pay more than $100. At retail, the solutions developed for this study ranged from a minimum of $100 (for the smallest HEPA indoor air cleaner deployed in smaller mobile homes) to about $400 (for 6 × 4” MERV-13 filters and a larger HEPA indoor air cleaner). Because there was no significant wildfire smoke event during the study period, we were unable to determine how long MERV-13 filters should be left on ECs to effectively filter the air entering homes. However, after leaving the filters on for several weeks, they had noticeable water damage and were infiltrated with dust. The filters are temporary solutions and periodic filter replacements would be a significant ongoing expense for participants.

Filter interventions could be subsidized through government or private-sector programs. For example, in California, the CalAIM Community Supports Program allows Medi-Cal managed care plans to provide supplies and services to remediate environmental asthma triggers, including HEPA air purifiers (29). Filters could also be provided through community-based nonprofit groups funded by charitable donations, or in-kind donations from companies, but such efforts are likely to be relatively small-scale. The California Energy Commission has a program that includes weatherization and upgrades in heating/cooling technology for residents in low-income communities (30). This type of program could be replicated in other regions with likely health benefits through reductions in exposure to particulate matter and extreme temperatures.

## Conclusion

Temporary filtration solutions are feasible to reduce indoor wildfire smoke exposure through ECs. However, such solutions will likely need to combine external filtration and use of a HEPA indoor air cleaner and would need to be offered in the community at low-or no-cost to reduce barriers to adoption. More permanent solutions would also require significant cost subsidies; these include prioritizing homes with ECs in wildfire smoke exposed regions for replacement with heat pump technology or air conditioning. While this solution would reduce water consumption, it could increase energy use relative to ECs.

## Data Availability

The datasets presented in this study can be found in online repositories. The names of the repository/repositories and accession number(s) can be found at: https://osf.io/arz3d/.
